# The Lester Tool : An audit of its use on an acute psychiatry inpatient wards.

**DOI:** 10.1192/j.eurpsy.2023.1888

**Published:** 2023-07-19

**Authors:** S. N. B. Che Azih, A. Turley, R. Amarasinghe, K. Raju

**Affiliations:** 1 Psychiatry, Cheshire and Wirral Partnership NHS Foundation Trust, MACCLESFIELD, United Kingdom

## Abstract

**Introduction:**

People with severe mental illness have an increased vulnerability to cardiovascular disease due to multilple biopsychosocial factors and a potential adverse effects of longer term treatment with anti-psychotic medications. Treatment of severe and enduring mental illness with antipsychotic medications is likely to cause metabolic changes leading to weight gain and dyslipidaemia, thus increasing risk of cardiovascular disease. Cardiometabolic risk screening can be done using Lester Tool which also provides recommendations for interventions.

**Objectives:**

To identify the compliance to Lester Tool in the monitoring of cardiometabolic risk factors and intervention provided on acute psychiatry inpatients.

**Methods:**

We carried out a retrospective audit of 30 patients on regular antipsychotic medication on an adult inpatient ward in Macclesfield, United Kingdom. Electronic records were reviewed to establish whether the smoking status, lifestyle, BMI, blood pressure, blood glucose and blood lipid were documented with evidence of interventions being provided.

**Results:**

Of all 30 patients, none had shown compliance to all the parameters within the Lester Tool. 100% of the smoking status was documented, amongst which 78% were provided with interventions. 7% has lifestyle and diet status documented, of which 50% were given dietary advice. 80% had BMI documented, amongst which none were provided with any intervention. 90% had blood pressure documented, of which 50% were given any intervention. 40% had blood glucose documented, of which all were provided with intervention. 57% had blood lipid documented, of which none were provided with any intervention.

**Conclusions:**

Our results have shown the need of further awareness on the usefulness of the Lester Tool in an acute inpatient ward. Our recommendation would be to regularly train and educate inpatient staff to ensure that all the necessary parameters be monitored and provided with interventions.

**Disclosure of Interest:**

None Declared

